# Type I interferons drive inflammasome-independent emergency monocytopoiesis during endotoxemia

**DOI:** 10.1038/s41598-017-16869-2

**Published:** 2017-12-05

**Authors:** Corentin Lasseaux, Marie-Pierre Fourmaux, Mathias Chamaillard, Lionel Franz Poulin

**Affiliations:** Univ. Lille, CNRS, INSERM, CHU Lille, Institut Pasteur de Lille, U1019 - UMR 8204 - CIIL - Center for Infection and Immunity of Lille, F-59000, Lille, France

## Abstract

Emergency monocytopoiesis is an inflammation-driven hematological process that supplies the periphery with monocytes and subsequently with macrophages and monocyte-derived dendritic cells. Yet, the regulatory mechanisms by which early bone marrow myeloid progenitors commit to monocyte-derived phagocytes during endotoxemia remains elusive. Herein, we show that type I interferons signaling promotes the differentiation of monocyte-derived phagocytes at the level of their progenitors during a mouse model of endotoxemia. In this model, we characterized early changes in the numbers of conventional dendritic cells, monocyte-derived antigen-presenting cells and their respective precursors. While loss of caspase-1/11 failed to impair a shift toward monocytopoiesis, we observed sustained type-I-IFN-dependent monocyte progenitors differentiation in the bone marrow correlated to an accumulation of Mo-APCs in the spleen. Importantly, IFN-alpha and -beta were found to efficiently generate the development of monocyte-derived antigen-presenting cells while having no impact on the precursor activity of conventional dendritic cells. Consistently, the LPS-driven decrease of conventional dendritic cells and their direct precursor occurred independently of type-I-IFN signaling *in vivo*. Our characterization of early changes in mononuclear phagocytes and their dependency on type I IFN signaling during sepsis opens the way to the development of treatments for limiting the immunosuppressive state associated with sepsis.

## Introduction

Sepsis is a relatively common, life-threatening syndrome in which a systemic bacterial infection triggers a dysregulated host inflammatory response and leading to an immunosuppressive state associated with the development of secondary and nosocomial infections^1–3^. Although the inflammatory response is often brought under control in the intensive care unit, the immunosuppressive state appears to increase subsequently the likelihood of death in sepsis patients^1,4^. Although specific antisepsis treatments and reliable sepsis biomarkers are still lacking^5^, dendritic cells (DCs) are considered to be crucial for the resolution of sepsis and to combat life-threatening infection^6–14^.

Notably, *Escherichia coli* is a major cause of sepsis in hospitalized patients^15^. The cell wall of *E. coli* contains lipopolysaccharide (LPS), which triggers the expression of type I interferon (IFN)^16^, upon its recognition by Toll-like receptor 4 (TLR4). Type I IFNs constitute a multigene family whose main members (IFNα and IFNβ) have a major role in mediating the lethal effects of septic shock^17–19^. Type I IFNs exert their biological effects by binding to at least two transmembrane receptors (Ifnar1 and Ifnar2) and thus activating intracellular pathways leading to the expression of various IFN regulated genes^20,21^. On one hand, type I IFNs are required for the successful resolution of infections. On the other, type I IFNs are harmful during endotoxemia^22^. This duality may explain why *in vivo* experiments in mouse models have prompted different conclusions about their involvement in sepsis^23^. Consequently, the type I IFNs’ exact role in sepsis has yet to be clearly defined. As most deaths in human sepsis occur during the prolonged period of immunosuppression that follows the acute inflammation, we used a murine model of non-lethal endotoxemia to determine the role of type I IFNs in emergency monocytopoiesis and in the decrease of conventional dendritic cells (cDCs). Due to the protective role of DCs during sepsis, some researchers have argued that maintaining DCs function should be a key objective in this field^12,24–27^.

There are several subsets of DCs, which originate from either monocytic precursors differentiating into monocyte-derived antigen-presenting cells (Mo-APCs) or from non-monocytic progenitors differentiating into cDCs^28,29^. Both lineages are generally studied by characterizing their surface markers; all the subsets display the integrin CD11c and major histocompatibility complex class II (MHCII). They are part of the mononuclear phagocyte lineage, which originate from the bipotent macrophage and DC progenitor (MDP). The latter can differentiate into either a common monocyte progenitor (cMoP)^30^ or a cDC precursor (CDP)^31^. The CDPs give rise to pre-DCs, which migrate from the bone marrow to produce cDCs in peripheral tissues^31^. The latter can be further divided into two subsets (namely cDC1 and cDC2)^28^, both of which can be generated by *in vitro* culture of bone marrow cells with the cDC-inducing growth factor FMS-related tyrosine kinase 3 ligand (Flt3-L)^32^. We and others have shown that the surface markers CD64 (also known as FcγRI) and MerTK are specific for Mo-APCs, allowing the distinction between such cells and cDC^28,33–36^. However, most of the studies in this area were performed before it became possible to distinguish between cDCs and monocyte-derived APCs with the marker CD64^28,29^. In this context, several inflammatory cytokines (such as IFNα) favor the proliferation of hematopoietic stem cells with a bias towards the myelomonocytic hematopoietic branch, although IFNα has also been described as an inhibitor of hematopoiesis^37^. Indeed, emergency monocytopoiesis is thought to modulate hematopoietic stem and progenitor cells (HSPCs) and non-self-renewing precursors that express TLR4^38^. Indeed, it has been suggested that TLR activation alters the function and fate of HSPCs^39^.

In the present study, we discriminated between cDCs and monocyte-derived APCs by gating on the monocytic lineage marker CD64^28,33,34^. We found that (i) cDCs and their precursors were impaired by a low-dose LPS injection, and (ii) LPS-induced induction of Mo-APCs and their precursors was dependent on type I IFN signaling in spleen and bone marrow. Moreover, we demonstrated that IFNα/β allows the generation of Mo-APCs from MDPs *in vitro*, without impairing cDC development. This knowledge of the upstream modulation of medullar monocytopoiesis and their dependency on type I IFN signaling is likely to facilitate the development of treatments that limit the immunosuppressive state associated with sepsis.

## Results

### LPS-induced endotoxemia is associated to the development of Mo-APCs in a type-I IFN dependent manner

**Figure 1:**
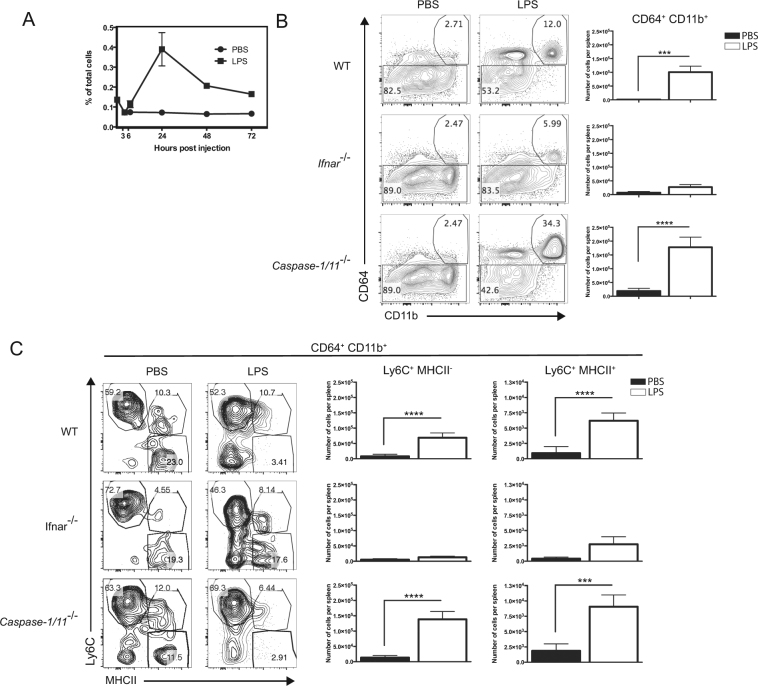
Induction of splenic monocyte-derived antigen presenting cells during LPS-induced endotoxemia depends on type I IFN. Wild-type (WT), *Ifnar1*-KO, and *Caspase-1/11*-KO mice were injected intravenously (IV) with a non-lethal dose of ultrapure LPS from *E. coli* O111:B4 (25 μg/mouse), or PBS. Spleen cells were analyzed by flow cytometry in kinetic in WT mice (**A**) or 24 h after LPS injection in Wild-type (WT), *Ifnar1*-KO, and *Caspase-1/11*-KO mice (**B** and **C**). Splenic Mo-APC were gated as Lin^−^ CD135^−^ CD11b^+^ CD64^+^ to quantified their number (**A**) and their expression of Ly6C and MHCII has been assessed (**B**). Data are representative of at least two independent experiments done in triplicates. Bars indicate mean ± SEM. Statistical significance was assessed by non-parametric Mann-Whitney test. P < 0.01 (**), P < 0.001 (***) and P < 0.0001 (****) were considered statistically significant.

To investigate the impact of LPS on the development of Mo-APCs in mice, we counted these APCs in the spleen at various time points after an intravenous (IV) injection of LPS (Fig. 1). Single-cell suspensions were prepared from the spleens and analyzed using multiparameter flow cytometry. Live singlet cells were gated on MHCII, and lineage-positive (Lin^+^) cells (such as T, B and NK cells, eosinophils and neutrophils) were excluded based on CD3, CD19, NK1.1, CCR3, and Ly6G, respectively^33^. Subsequently, Lin^−^ (lineage-negative) MHCII^+^ cells were divided into cDCs and Mo-APCs, based on the latter’s expression of CD64 and non-expression of the cDC marker CD135^28,33^ (Fig. 1A). The Mo-APC count had increased significantly 24 h after an IV injection of LPS (Fig. 1A). Given that hematopoietic progenitor cells can respond to inflammatory cytokines like IFNα and IL-1^40,41^, mice deficient for Ifnar1 (*Ifnar1* ^−/−^) or for Caspase-1/11 (*Casp1/11* ^−/−^) were injected with ultrapure LPS or PBS only, and their spleen harvested at 24 h. In contrast to *Casp1/11* ^−/−^ mice, LPS-induced Mo-APCs were not observed in *Ifnar1*-deficient mice (Fig. 1B) and also in the bone marrow of these mice (see Supplementary Figure S1). Meanwhile required for LPS-induced IL-18 augmentation in the serum (see Supplementary Figure S2A), Caspase-1/11 expression was dispensable for the increased proportion of Mo-APC**s** in the bone marrow and spleen following LPS injection. Additionally, bone marrow cells deficient for Asc (referred herein as *Pycard* ^−/−^ mice) are not affected in their ability to generate Mo-APCs in the presence of Flt3-L and LPS (see Supplementary Figure S2B). These data indicate that the inflammasome is dispensable for the type-I-IFN-dependent increase in Mo-APC counts. We further analyzed the phenotype of the Mo-APC cells induced 24 h after LPS injection. The CD64^+^ CD11b^+^ induced cells have a phenotype reminiscent of the so-called monocyte-waterfall. Briefly, recruited monocytes during inflammation acquire MHCII, and CD64 expression, and lose progressively the marker Ly6C^33^. We observed a significant increase in the population CD64^+^ CD11b^+^ Ly6C^+^ after LPS treatment regardless of MHCII expression in WT and *Casp1/11* ^−/−^ mice (Fig. 1C), and also in the bone marrow of these mice (see Supplementary Figure S1). These results argue for a monocytic origin of the CD64^+^ cells induced after systemic LPS treatment^33^. Altogether, these results indicate that LPS-induced endotoxemia is associated to the induction of Mo-APCs in a type-I IFN manner, independently of inflammasome activation.

### LPS-induced endotoxemia impaired conventional DCs development

**Figure 2:**
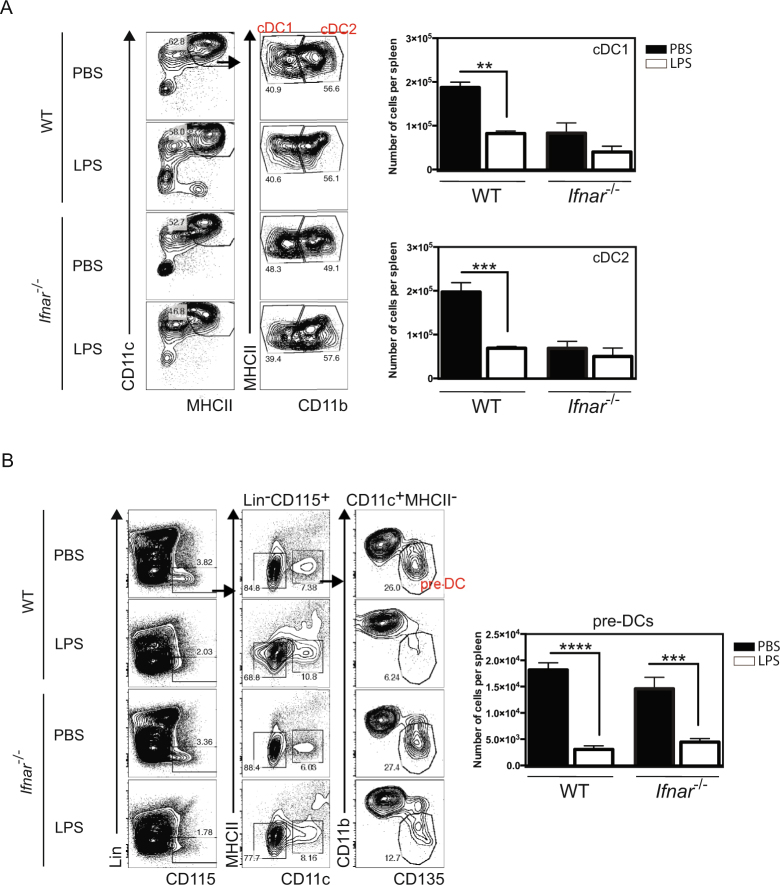
Decrease of splenic conventional dendritic cells numbers and their precursors in bone marrow during endotoxemia. WT and *Ifnar1*-KO mice were treated as described in Fig. 1. Spleens and bone marrows were collected and analyzed by flow cytometry 24 h after LPS injection. Dendritic cells (DCs) were gated as Lin^−^ CD64^−^ MHCII^+^ CD11c^+^ in the spleen and were divided in cDC1 and cDC2 based on the CD11b expression on the latter (**A**) and bone marrow pre-DC were gated as Lin^−^ CD115^+^ CD11c^+^ MHCII^−^ CD135^+^ CD11b^−^ (**B**) to quantify their number. Bars indicate mean ± SEM from 3 independent experiments. Statistical significance was assessed by non-parametric Mann-Whitney test. P < 0.01 (**), P < 0.001 (***) and P < 0.0001 (****) were considered statistically significant.

Spleen DCs (Lin^−^ CD64^−^ MHCII^+^ CD11c^+^) were divided in cDC1 and cDC2 based on the CD11b expression on the latter (Fig. 2A). Both DC populations were significantly reduced 24 h after LPS treatment in WT mice (relative to control mice injected with PBS only). The lower number of cDCs in the spleen of untreated *Ifnar1* knock-out (KO) mice impeded any conclusion on the LPS effect on the development of these cells (Fig. 2A, lower panel). The lower cDC number in *Ifnar1*-KO is not due to the gating strategy as we take into account the putative lower MHCII level reported in these mice due to the role of type I IFN on cDC maturation^42,43^ by taking not only the MHCII high cells but also the intermediate ones. We concluded that the increase in the Mo-APC count was accompanied by a decrease in the cDC number. In order to study this mechanism in more details, we counted the numbers of direct cDC precursors (namely pre-DCs) in the bone marrow as early as 24 h after the LPS injection. The pre-DC was gated as Lin^−^ CD115^+^ CD11c^+^ MHCII^−^ CD135^+^ live singlet cells^8^ (Fig. 2B). In control animals, the absolute count of pre-DCs in the bone marrow was significantly reduced 24 h after LPS injection; this observation is consistent with a decreased number of cDCs during LPS-mediated inflammation (Fig. 2A). To establish whether this affected pre-DC count following LPS treatment were dependent on type I IFNs, we counted numbers of pre-DCs within the bone marrow of *Ifnar1*-KO mice after LPS injection. We found that the decrease of LPS-induced pre-DCs in bone marrow is type I IFN independent. These results argue for a reduction of cDCs and their pre-DC precursors during LPS-induced endotoxemia.

### LPS-induced endotoxemia stimulated monocytopoiesis

**Figure 3:**
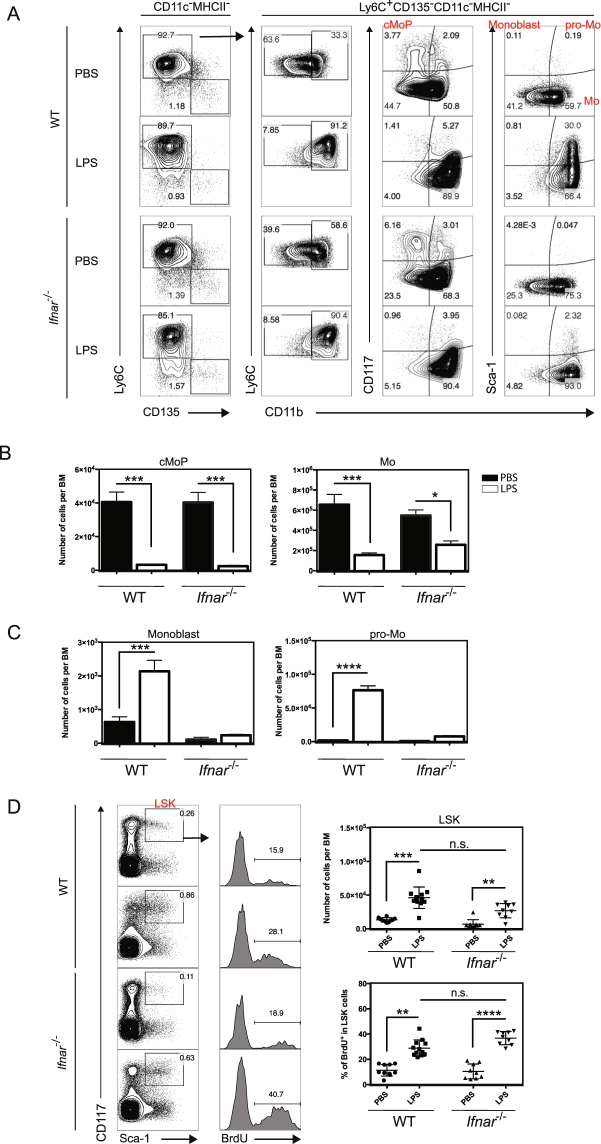
Monocytopoiesis during LPS-induced endotoxemia depends on type I IFN signaling. WT and *Ifnar1*-KO mice were treated as described in Fig. 1. Bone marrows were collected and analyzed by flow cytometry 24 h after LPS injection. Among Lin^−^ CD115^+^ CD11c^−^ MHCII^−^ bone marrow cells, cMoP were gated as Ly6C^+^ CD135^−^ CD11b^−^ CD117^+^, monoblasts as Ly6C^+^ CD135^−^ CD11b^−^ Sca1^+^, pro-monocytes (pro-Mo) were gated as Ly6C^+^ CD135^−^ CD11b^+^ Sca1^+^, monocytes (mono) as Ly6C^+^ CD135^−^ CD11b^+^ Sca1^−^ and MDP as Ly6C^-^ CD135^+^ CD117^+^ (**A**), and their number were calculated (**B**,**C**). Data are representative of 3 independent experiments (**A**,**B** and **C**). Bars indicate mean ± SEM from 1 (**A**,**B** and **C**). Statistical significance was assessed by non-parametric Mann-Whitney test. P < 0.05 (*), P < 0.01 (**), P < 0.001 (***) and P < 0.0001 (****) were considered statistically significant.

To determine whether the LPS-mediated induction of Mo-APC**s** is correlated with an induction of monocytopoiesis, we counted the recently described monocyte committed progenitors (namely cMoP) 24 h after LPS injection. The cMoP was gated as described previously^8,30,44^. Briefly, live singlet Lin^−^ CD115^+^ CD11c^−^ MHCII^−^ Ly6C^+^ cells (Fig. 3A, left) were analyzed for Ly6C vs. CD11b, CD117 vs. CD11b, or Sca-1 vs. CD11b, in order to distinguish between cMoPs (live singlet Lin^−^ CD115^+^ CD11c^−^ MHCII^−^ Ly6C^+^ CD117^+^ CD11b^−^ cells), and monocytes (live singlet Lin^−^ CD115^+^ CD11c^−^ MHCII^−^ Ly6C^+^ Sca-1^−^ CD11b^+^ cells) (Fig. 3A). At 24 h after LPS injection, monocytes were significantly decreased in the bone marrow (Fig. 3A and B) and in the blood (see Supplementary Figure S3) of WT and *Ifnar1*-KO mice. This drop of monocytes from the bone marrow might reflect a higher recruitment of these cells towards the peripheral organs (such as the spleen) to favor the generation of LPS-induced Mo-APC**s**. To determine the effects of LPS on cMoPs, bone marrow cells were counted in WT mice after an injection of LPS or PBS. As reported previously in the context of bacterial infection^45^, a significant drop in the number of cMoP precursor cells was observed 24 h after LPS injection (Fig. 3A and B). These observations indicate that LPS rapidly induces a loss of cMoPs. To determine whether this decrease in cMoPs resulted from accelerated differentiation into monoblasts and promonocyte (pro-Mo) cells (as suggested by^45^), we counted these precursors in the bone marrow. Monoblasts were defined as live singlet Lin^−^ CD115^+^ CD11c^−^ MHCII^−^ Ly6C^+^ Sca-1^+^ CD11b^−^ cells, and pro-Mo cells were defined as live singlet Lin^−^ CD115^+^ CD11c^−^ MHCII^−^ Ly6C^+^ Sca-1^+^ CD11b^+^ cells^45^. We detected a significant increase in the number of monoblasts and pro-Mo cells within the bone marrow after LPS injection when compared to PBS injection (Fig. 3A and C). This observation indicates that LPS treatment induces monocytopoiesis in the bone marrow. Given that cMoP cells express both Ifnar1 and Ifnar2^30^, we next determined the impact of LPS injection on monocytopoiesis in *Ifnar1*-deficient mice. As had been observed in controls, we found that the cMoP count in the bone marrow of *Ifnar* ^−/−^ mice had decreased 24 h after LPS injection. Although LPS-induced fall in the cMoP count was Ifnar independent (Fig. 3A and B), LPS-induced increase in monoblast and pro-Mo counts was Ifnar dependent (Fig. 3A and C). As positive and negative effects of type-I IFN on HSPCs are described in the literature^37^, we measured the proliferation and number of LSK bone marrow cells (Lin^−^ Sca-1^+^ c-kit^+^)^45,46^. LSK cells were gated as described in Supplementary Figure S4A ^45^, and a significant increase of their number and proliferation, evaluated by BrdU incorporation, was observed in a type-I IFN independent manner (see Supplementary Figure S4B). Then, LPS induced the generation of monocyte precursors 24 h after its injection in a type-I dependent manner. These observations indicates that the LPS-dependent increase in the monoblast and pro-Mo counts in the bone marrow was type I IFN signaling dependent. This finding indicates that LPS-induced monocytopoiesis requires intact Ifnar1 signaling.

### LPS induced Mo-APCs on macrophage/dendritic cell precursor in type-I IFN dependent manner

**Figure 4:**
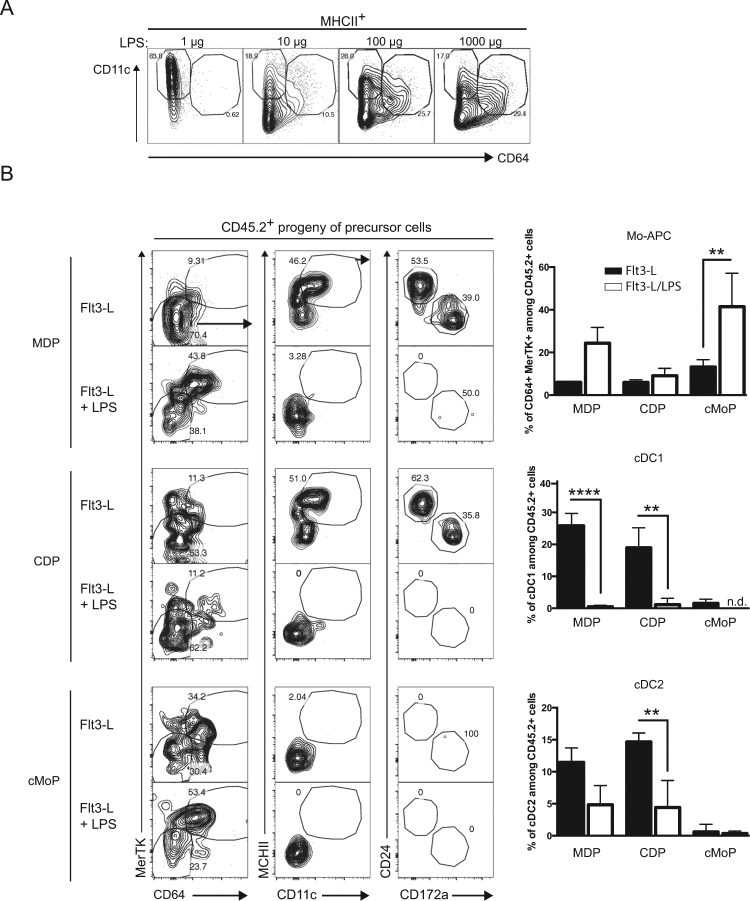
LPS induced Mo-APCs are derived from monocyte progenitors *in vitro* but not from pre-DC. Increasing doses of LPS ultrapure from *E. coli* O111:B4 were added at day 0 in Flt3-L-dependent dendritic cells culture. After 7 days, the Flt3-L-treated bone marrow cells were analyzed by flow cytometry for the presence of DC (gated as MHCII^+^ CD11c^+^ CD64^−^) and Mo-APC (gated as MHCII^+^ CD11c^+^ CD64^+^) (**A**). CD45.2^+^ cMoP (gated as Lin^−^ MHCII^−^ CD11c^−^ CD115^+^ CD135^−^ CD117^+^ Ly6C^+^ CD11b^−^), CDP (gated as Lin^−^ MHCII^−^ CD11c^−^ CD115^+^ CD135^+^ CD117^−^ Ly6C^−^ CD11b^−^) and MDP (gated as Lin^−^ MHCII^−^ CD11c^−^ CD115^+^ CD135^+^ CD117^+^ Ly6C^−^ CD11b^−^) were sorted by flow cytometry and were co-cultured with CD45.1^+^ bone marrow filler cells in Flt3-L-dependent dendritic cells culture. Cultures were supplemented with LPS (100 ng/ml) at day 0 and the cDC1 (gated as CD64^−^ MerTK^−^ MHCII^+^ CD11c^+^ CD24^+^ CD172a^−^), cDC2 (gated as CD64^−^ MerTK^−^ MHCII^+^ CD11c^+^ CD24^−^ CD172a^+^) and Mo-APC (gated as CD64^+^ MerTK^+^) composition after 7 days was measured. The frequencies of each population among the CD45.2^+^ cells were calculated (**B**). Data are from one experiment done in triplicate. Bars indicate mean ± SEM. Statistical significance was assessed by non-parametric Mann-Whitney test. P < 0.01 (**) were considered statistically significant.

**Figure 5:**
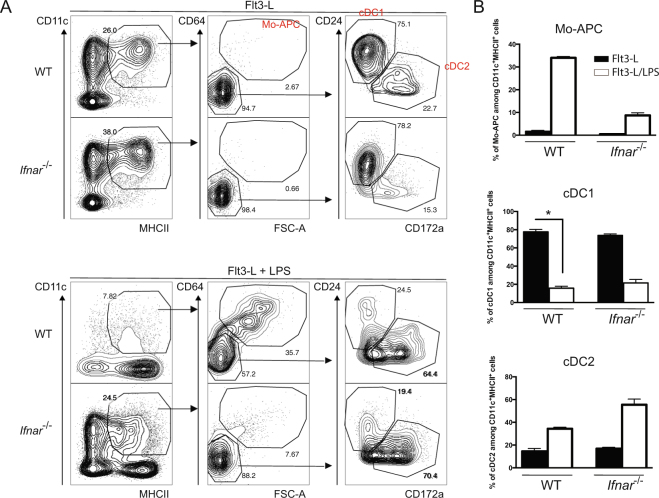
Type I IFN signaling is required for LPS-induced Mo-APC *in vitro*. LPS ultrapure from *E. coli* O111:B4 (100 ng/ml) were added or not at day 0 in Flt3-L-dependent *in vitro* dendritic cells cultures generated with WT or *Ifnar1*-KO bone marrow cells. After 7 days, the Flt3-L-treated bone marrow cells were analyzed by flow cytometry for the presence of cDC1 (gated as MHCII^+^ CD11c^+^ CD64^−^ CD24^+^ CD172a^−^), cDC2 (gated as MHCII^+^ CD11c^+^ CD64^−^ CD24^−^ CD172a^+^) and Mo-APC (gated as MHCII^+^ CD11c^+^ CD64^+^) (**A**). The frequencies of these populations were calculated for each condition (**B**). Data are representative of at least 2 independent experiments done in quadruplicate. Bars indicate mean ± SEM. Statistical significance was assessed by one-way ANOVA/Bonferroni posttest. P < 0.05 (*), P < 0.01 (**), P < 0.001 (***) and P < 0.0001 (****) were considered statistically significant.

With a view to establish whether LPS induces monocytopoiesis by modulating the development of bone marrow cells, we studied *in vitro* cultures of Flt3-L-derived DCs^32^. To establish whether LPS can induce Mo-APC**s** by modulating progenitor development in Flt3-L-DCs, we titrated the induction of Mo-APCs (live singlet MHCII^+^ CD11c^+^ CD64^+^ cells) in response to increasing concentrations of LPS in the culture. We found that LPS concentrations ranging from 10 to 1000 ng/ml induced Mo-APCs (Fig. 4A). To confirm the monocytic origin of these *in vitro* generated Mo-APCs, we sorted MDP, CDP and cMoP and cultured them on filler cells in the presence of Flt3-L or Flt3-L and LPS to measure the origin of the induced Mo-APC**s**. As expected only MDP and cMoP cells were able to generate Mo-APC**s** in the presence of LPS (Fig. 4B). These results showed that LPS addition during *in vitro* cultures of Flt3-L-derived DCs induced Mo-APC**s**. To determine whether type I IFN signaling is required for the generation of LPS-induced Mo-APCs in bone marrow cells, we compared *Ifnar1*-deficient and WT bone marrow cells cultured with Flt3-L in the presence of LPS (Fig. 5A and B). Flt3-L-DCs were analyzed in order to determine the proportions of Mo-APCs (live singlet MHCII^+^ CD11c^+^ CD64^+^ cells), cDC1s (live singlet MHCII^+^ CD11c^+^ CD64^−^ CD24^+^ CD172a^−^ cells) and cDC2s (live singlet MHCII^+^ CD11c^+^ CD64^−^ CD24^−^ CD172a^+^ cells) (Fig. 5A). As expected, we found very few Mo-APCs in the Flt3-L-DC culture (Fig. 5A, upper part). However, cDC1s and cDC2s were present in both WT and *Ifnar1*-deficient bone marrow (Fig. 5A, upper part). In contrast to experiments with Flt3-L alone, the addition of LPS to the Flt3-L-bone marrow culture at day 0 was associated with a significant increase in the number of Mo-APCs (Fig. 5A, lower part). These results indicate that early addition of LPS to Flt3-L-bone marrow culture system makes the latter a good model of LPS-induced monocytopoiesis. To establish whether type I IFN signaling is required for LPS-induced monocytopoiesis, *Ifnar1*-deficient bone marrow cells were compared with WT bone marrow cells in a LPS-Flt3-L-bone marrow culture (Fig. 5A, lower part). We observed that *Ifnar1*-deficient bone marrow cells produced fewer Mo-APCs, which is consistent with a crucial role of type I IFN in LPS-induced monocytopoiesis.

**Figure 6:**
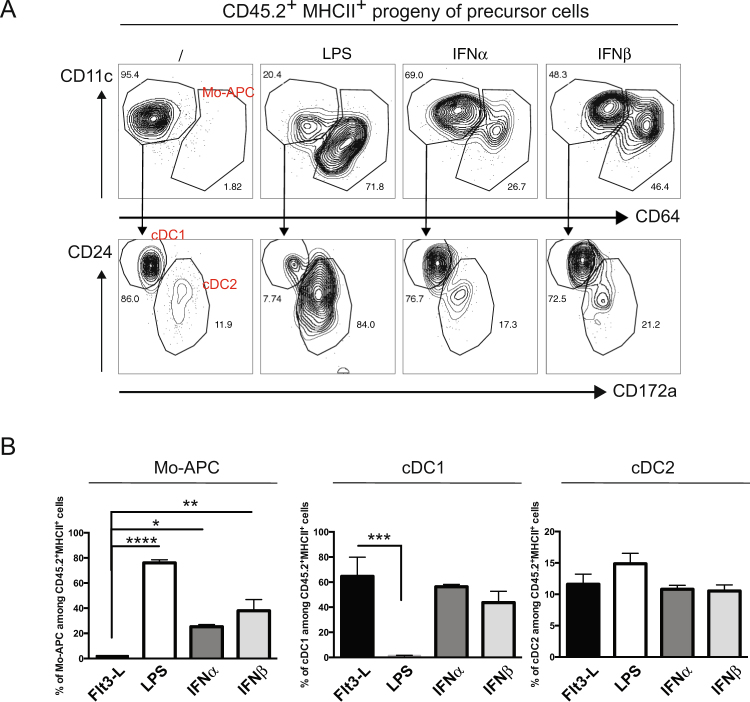
IFNα and IFNβ favour macrophage and DC progenitor development toward monocyte-derived antigen presenting cells. CD45.2^+^ MDP (gated as described in Fig. 3) sorted by flow cytometry were co-cultured with CD45.1^+^ bone marrow filler cells at day 0 in Flt3-L-dependent dendritic cells cultures. Cultures were supplemented with LPS (100 ng/ml), IFNα (100 ng/ml) or IFNβ (10 ng/ml) at day 0 and the DC and Mo-APC composition after 7 days was measured by flow cytometry (**A**). The frequencies of each population among the CD45.2^+^ MHCII^+^ cells were calculated (**B**). Data are representative of at least 2 independent experiments done in quadruplicate. Bars indicate mean ± SEM. Statistical significance was assessed by one-way ANOVA/Bonferroni posttest. P < 0.05 (*), P < 0.01 (**), P < 0.001 (***) and P < 0.0001 (****) were considered statistically significant.

To establish whether type I IFN is able to influence MDP progenitors and favor their differentiation into Mo-APCs, MDPs from CD45.2^+^ mice were sorted and cultured on CD45.1^+^ filler cells^44^ in the presence of Flt3-L and in the presence or absence of IFNα, IFNβ or LPS (Fig. 4C). We gated on the progeny of the precursor cells by selecting live singlet CD45.2^+^ MHCII^+^ cells (Fig. 6A) and analyzed frequencies of Mo-APCs, cDC1s and cDC2s after 7 days of culture (Fig. 6B). As expected, purified MDP donor cells gave rise to only cDC1s and cDC2s in the presence of Flt3-L. The addition of IFNα or IFNβ at day 0 of the Flt3-L-DC culture induced a significant increase in Mo-APC counts. Addition of LPS favored Mo-APC induction and impaired cDC generation; in contrast, addition of IFNα/β was not associated with a decrease in cDC differentiation despite Mo-APC**s** generation. These observations indicate that IFNα/β acts on bone marrow cells to drive the generation of MDP-derived Mo-APCs. More precisely, we hypothesize that MDPs and cMoPs might be direct targets of type I IFN, as both Ifnar1 and Ifnar2 are expressed (see Supplementary Figure S5A). To test this hypothesis, MDPs and cMoP from CD45.2^+^ WT or *Ifnar1*-deficient mice were sorted and cultured on filler cells as described above and their ability to generate Mo-APC cells was measured, and the presence of filler-derived Mo-APCs was used as an internal control (see Supplementary Figure S5B). The presence of LPS as expected favored Mo-APC**s** on filler cells and WT precursors; in contrast, the *Ifnar1*-deficient MDP or cMoP were impaired in their ability to produce Mo-APCs, which is coherent with an intrinsic role of Ifnar1 signaling on monocyte precursors during LPS-induced monocytopoiesis.

## Discussion

**Figure 7:**
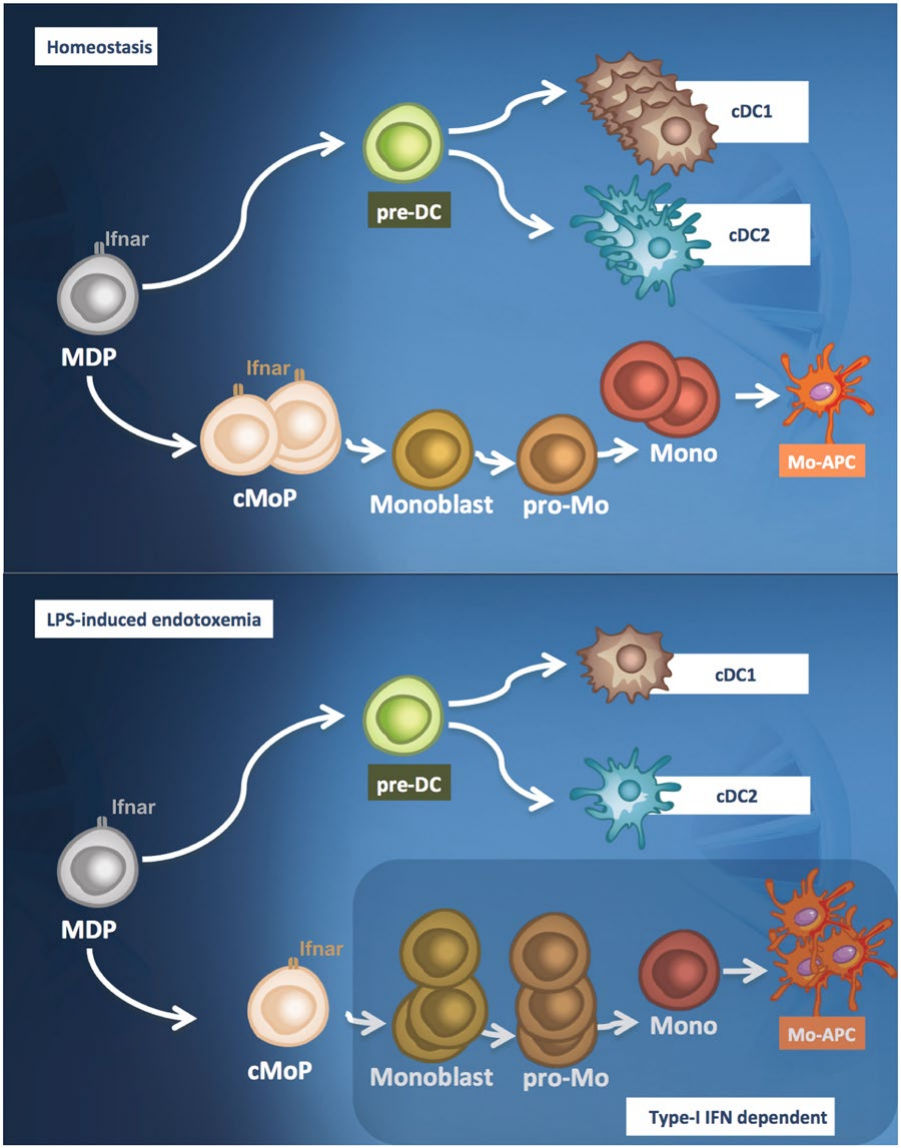
Schematic overview of the mechanisms leading to Mo-APC induction during LPS-induced endotoxemia. In the steady state, the myeloid progenitors develop into conventional dendritic cells or Mo-APCs (upper part). LPS-induced endotoxemia favors a type-I IFN dependent monocytopiesis at the expense of conventional DCs generation (lower part). cDC conventional dendritic cell; LPS lipopolysaccharide; MDP macrophage and DC progenitor; pre-DC precursor of DCs; cMoP common monocyte progenitor; pro-Mo promonocyte; mono monocytes; Mo-APC monocyte-derived antigen-presenting cells.

Here, we demonstrated that LPS induces monocytopoiesis in a type-I-IFN-dependent manner. This correlates with a decrease in cDCs and their precursors. Furthermore, we showed for the first time that type I IFN, IFNα and IFNβ, modulate the fate of MDP/cMoP and increase monocytic progeny. Our results indicate that type I IFN signaling in an inflammatory environment favors the generation of immune cells (Fig. 7).

Endotoxemia is generally associated by a decrease in the cDC count^6–11^. The cDC count is decreased directly by inducing cell death^1,9,24,47^. For instance, inhibition of apoptotic mediator such as caspase-8 increases mice resistance in sepsis model^48^. Alternatively, cDCs count might also be affected by decreasing cDC generation^45^. For example, the cDC content decreases after a bacterial infection as a result of a decrease in the number of pre-DC precursors, with no change in the level of apoptosis^45^. Here, by using the discriminating marker CD64 and a murine model of endotoxemia, we noted a rapid increase in the Mo-APC count and a decrease of cDCs numbers in spleens^33,34^. Indeed, we observed a decrease in splenic cDCs counts after *in vivo* LPS treatment, which is consistent with the reduction in the pre-DCs numbers in bone marrow. Moreover, *in vitro* addition of LPS induced a similar decrease in cDCs composition of Flt3-L-derived DCs. This LPS-induced reduction in cDCs is counterbalanced by a type I IFN dependent generation of Mo-APCs. The exact mechanism by which LPS reduced pre-DC precursors and cDCs requires more future analysis.

Induction of monocytopoiesis has been reported in various sepsis-related models. In mice, the monocytopoiesis induced by bacterial infections (e.g. with *Yersinia enterocolitica*) is similar to that observed upon LPS treatment^45^. In fact, the cMoP count decreases rapidly in a TLR4- and IFNγ-dependent manner, leading to high numbers of Sca-1^+^ monoblasts and promonocytes^45^. Similarly, LPS induces a decrease in numbers of the upstream MDP precursor, namely the granulocyte-macrophage progenitor^49^. Moreover, monocytopoiesis during *Listeria monocytogenes* infection is characterized by a significant, Caspase-1-independent increase in the number and proliferation of monocytes from the bone marrow^50^. Finally, monocytopoiesis is also induced during *E. col*i-infected mice by the accumulation of mobilized HSPCs in the spleen^51^.

The exact source of type I IFN remains to be identified with a particular attention on plasmacytoid DCs (pDCs) which are primarily secreting type I IFN in several pathological conditions^52–54^. Aside from its role in secreting type I IFNs during endotoxemia, pDCs might also be critical cells regulating endotoxemia through their function in cross-priming and cross-presentation of antigen to T cells^55–57^. Meanwhile, recent single cell data revealed that antigen presenting functions of pre-DCs were wrongly attributed to pDC^58,59^, highlighting the necessity to revisit not only the definition of pDCs but also their role during endotoxemia and sepsis. Moreover, we cannot exclude that LPS-induced monocytopoiesis may account for the activation of pDCs by type I interferons^60^. However, monocyte precursors cell intrinsic Ifnar signaling is required during LPS-induced monocytopoiesis to favor Mo-APC development.

Hematopoietic cells (including HCS and myeloid precursors) can be considered as targets for type I IFN. Indeed, type I IFN acts on hematopoietic cells and is required for survival in a mouse CLP-based sepsis model by increasing CXCL10 production, recruiting neutrophils and macrophages, and stimulating phagocyte functions^23^. Moreover, we found that LPS and IFNα/β were capable of inducing *ex vivo* Mo-APCs in the bone marrow, which predominantly contains hematopoietic cells, and that MDP and cMoP could be direct targets of type I IFN.

Aside a direct effect of type I IFN on MDP and cMoP, another target cell of type I IFN is monocyte, which responds to this trigger by producing IL-18 during viral infection^61^. However, we cannot rule out the possibility that type I IFN modulates progenitor cells indirectly by inducing other factors. With regard to type I IFN synergistic factors, IFNγ induces the differentiation of myeloid precursors and a decrease in the generation of neutrophils (in viral infections)^62^ or DCs (in bacterial infections)^45^. Similarly, IFNγ induces IL-27 production during malaria infection; IL-27 then promotes the expansion and differentiation of long term hematopoietic stem cells (HSCs) into myeloid progenitors, in synergy with stem cell factor (a c-kit ligand)^63^. In a mouse model of acute abdominal sepsis, IL-3 produced by B cells promotes a cytokine storm by inducing the differentiation of Ly6C^hi^ monocytes and neutrophils^64^. Blocking IL-3 production reduces the intensity of sepsis by decreasing inflammation-associated myelopoiesis^64^. Interestingly, IFN-I-activated B cells are protective in early innate immune responses during bacterial sepsis^65^.

Unexpectedly, we observed a significant decrease of cMoP counts at 24 h after LPS injection in bone marrow; this apparently contradicts the type-I-IFN-dependent increase in the bone marrow content of Sca-1-expressing monoblast, promonocyte precursor cells and LSK cells. Given that IFNα induces Sca-1 expression in HSPCs^66^, type I IFN signaling might modulate a rapid transition from Sca-1-negative cells to Sca-1 positive cells. However, we saw no impact of *Ifnar1* deficiency on LSK cell number and proliferation. Suggesting that our results can not be explained by a decreased Sca-1 expression or HSPC proliferation in *Ifnar1*-deficient mice. Sca-1 is not only a widely used HSPC marker but is also required for HSPC self-renewal and the development of committed progenitor cells^67^. Along the same lines, Sca-1 has a crucial role during severe bacterial infections in mice by diverting early hematopoietic precursors towards the myeloid lineage^68^. Moreover, HSPCs lacking Sca-1 (like those lacking the Ifnar) are insensitive to IFNα stimulation^37^; this observation demonstrates that Sca-1 mediates the IFNα-induced proliferation of HSPCs.

We hypothesize that in an inflammatory context (such as that created by exposure to LPS), type I IFN drives emergency monocytopoiesis by increasing the monocytic output of MDPs. Our present results show that exposure to LPS (a surrogate of bacterial septicemia) leads to type-I-IFN dependent monocytopoiesis by favoring the differentiation of MDPs into Mo-APCs. Although type-I-IFN-dependent monocytopoiesis might represent a potential escape mechanism for viruses^69^, it may enable the host to contain the invading pathogen by increasing the availability of innate immune cells. Meanwhile the role of type-I IFN is opposite in endotoxemia^22^ and sepsis models^23^, our observations might be applicable to other biological situations in which overproduction of type I IFN production is observed, such as viral infections and interferon-related diseases^54,70^.

Furthermore, we suggest that our findings might also apply to other CD11c-expressing cells, such as regulatory DCs (which expand during endotoxic shock^71^) and inflammatory DCs^72^. Our study opens up opportunities for detailed analyses of type-I-IFN-dependent monocytopoiesis in various inflammatory settings. Although monocytopoiesis is detrimental in the early acute sepsis phase (due to an enhanced inflammatory state), it is beneficial in the late immunosuppressive phase^73^. Based on our results in the mouse, we suggest that the cDC/Mo-APC content in septic patients should be re-evaluated. Unfortunately, the CD64 marker is not discriminative for human cDCs, although other gating strategies have been recently proposed^28^. Moreover, our *in vitro* model of progenitors cultured on filler cells might be a useful tool for determining the mechanism by which type I IFNs acts on progenitors to favor monocytopoiesis. In fact, the culture system dissociates the contrasting positive and negative effects of type I IFN on HSPCs without affecting the modulation of downstream targets like MDPs and cMoPs^37^. The molecular mechanisms by which type I IFNs render the host more vulnerable to secondary bacterial challenge (including exposure to other PAMPs such as bacterial muramyl dipeptide) merit further study. In summary, our findings describe the molecular mechanism of endotoxemia-associated monocytopoiesis and thus open up new perspectives for immunotherapeutic strategies in the fight against systemic microbial infections. For example, treatment with IFNα might restore normal monocytopoiesis and reduce susceptibility to secondary infections and/or the persistence of some viruses. Similarly, administration of anti-IFNα relieve monocyte-dependent inflammatory disorders.

## Materials and Methods

### The murine model, and induction of LPS-induced endotoxemia

C57BL/6 J mice (from Janvier Labs), *Caspase-1/11* ^−/−^^74^ and *Ifnar1* ^−/−^^75^ ((F. Trottein (CIIL) (housing) and B. Ryffel (CNRS, Orléans) (gift)) mice at 8 to 16 weeks of age received a retro-orbital, intravenous (IV) injection of 25 μg of LPS (O111:B4 Ultrapure, Invivogen) in 100 μl of Dulbecco’s PBS. Control mice received Dulbecco’s PBS only. Spleen, blood and bone marrow (femur and tibia) samples were collected at the indicated time points. The local investigational review board approved all animal studies (CEEA – “75 Comité d’Ethique en Expérimentation Animale Nord - Pas de Calais” (CEEA232009R). Animal experiments were performed in an accredited establishment (N° B59–108) according to governmental guidelines N°86/609/CEE.

### Cell preparation and flow cytometry

Bone marrow cells were flushed out of the bones. A single-cell suspension was prepared by repeated pipetting. Spleen samples were disaggregated by 30 minutes of 1 mg/ml Collagenase D (Roche) treatment and a single-cell suspension was prepared by repeated pipetting. Red blood cells were lysed by treatment with 160 mM NH_4_Cl and 170 mM Tris. Single-cell suspensions were incubated in the dark with LIVE/DEAD reagent (Thermo Fisher Scientific) for 30 minutes on ice. The cells were then incubated for 10 minutes with purified rat anti-mouse CD16/CD32 (Biolegend, 93 clone) and normal mouse serum (Interchim) before being stained with various monoclonal antibodies for 20 minutes in the dark on ice. Blood has been sampled in heparinized tubes by cardiac puncture immediately after sacrifice. Whole blood cells were then directly incubated with the antibodies for 20 minutes at room temperature in the dark. Red blood cells were lysed after staining with Optilyse B erythrolytic reagent (Beckman Coulter). Samples were analyzed with a LSR Fortessa flow cytometer (BD Biosciences) or sorted on a BD FACS Aria (BD Biosciences). The data were analyzed with Flowjo software V10.1 (TreeStar). The following antibodies were used for staining (Biolegend): PerCP anti-mouse CD3 (17A2), CD19 (6D5), NK1.1 (PK136), Ly6G (1A8) and Ter119 (TER-119), APC-Cy7 anti-mouse CD11b (M1.70), APC anti-mouse CD115 (AFS98) and CD64 (X54-5/7.1), PE anti-mouse CD135 (A2F10) and CD64 (X54-5/7.1), BV605 anti-mouse Sca1 (D7), Alexa Fluor 700 anti-mouse Ly6C (HK1.4), PeCy7 anti-mouse CD117 (2B8) and CD24 (M1/69), BV711 anti-mouse CD64 (X54-5/7.1), BV510 or FITC anti-mouse I-A/I-E (M5/114.15.2), and FITC anti-mouse CD172a (P84), and anti-mouse BrdU (3D4). The PE-CF594 anti-mouse CD11c (HL3) antibody was purchased from BD Biosciences.

### Serum and ELISA

Serums were harvested from blood samples, collected by cardiac puncture in heparinized tubes after sacrifice. For IL-18 ELISA, purified anti-IL-18 (Clone 74, MBL International) was used for coating the plates and biotin anti-IL-18 (Clone 93–10C, MBL International) was used for IL-18 detection. For standard curve, recombinant murine IL-18 (R&D system, B002-5) was used.

### Gene expression

Isolated RNA was reverse-transcribed with the cDNA synthesis kit (Agilent Technologies), according to the manufacturer’s instructions. The resulting cDNA (equivalent to 500 ng of total RNA) was amplified using the SYBR Green real-time PCR kit and detected on a Stratagene Mx3005 P (Agilent Technologies). qPCR was performed using forward and reverse primers (sequences available upon request). On completion of the PCR amplification, a DNA melting curve analysis was carried out in order to confirm the presence of a single amplicon. Actb was used as an internal reference gene in order to normalize the transcript levels. Relative mRNA levels (2-DDCt) were determined by comparing (a) the PCR cycle thresholds (Ct) for the gene of interest and Actb (DCt) and (b) DCt values for precursor cells and monocyte control group (DDCt).

### BrdU incorporation and intracellular staining

Single-cell suspensions of bone marrow cells were incubated for 1 hour *in vitro* with 10 μM BrdU in complete medium^76^. The cells were then harvested, washed and stained for extracellular markers as described previously. Intracellular BrdU staining was performed using a BrdU Flow Kit (BD Pharmingen), according to the manufacturer’s instructions.

### *In vitro* culture of bone marrow-derived DCs

Bone marrow cells were cultured with recombinant human Flt3-L (Celldex) as previously described^32^ and supplemented with 100 ng/ml LPS 0111:B4 Ultrapur (Invivogen), 100 ng/ml IFNα (Peprotech), 10 ng/ml IFNβ (Peprotech), or medium only. Cells were harvested, stained and analyzed by flow cytometry on day 7.

### Data availability

No datasets were generated or analyzed during the current study.

## Electronic supplementary material

Supplementary Information
