# Supplementary material for: Type I interferons drive inflammasome-independent emergency monocytopoiesis during endotoxemia
Source: Sci Rep. 2017 Dec 5;7:16935. doi: 10.1038/s41598-017-16869-2 (PMC5717267; doi:10.1038/s41598-017-16869-2)
Supplement: Supplementary file 1 — Supplementary Information [file 41598_2017_16869_MOESM1_ESM.pdf]

Supplementary Information

Type I interferons drive inflammasome-independent emergency monocytopoiesis during endotoxemia

Corentin Lasseaux[1], Marie-Pierre Fourmaux[1], Mathias Chamaillard[1], Lionel Franz Poulin[1]
[1]Univ. Lille, CNRS, INSERM, CHU Lille, Institut Pasteur de Lille, U1019 - UMR 8204 - CIIL - Center for Infection and Immunity of Lille, F-59000 Lille, France

Figure S1. Type I IFN dependent induction of bone marrow monocyte-derived antigen presenting cells during LPS-induced endotoxemia. Wild-type (WT), *Ifnar1*-KO and *Caspase-1/11*-KO mice were treated as described in Figure 1. Bone marrow cells were collected and analyzed by flow cytometry 24h after LPS injection. Mo-APC were gated as Lin^{-} $CD135^{-}$ $CD11b^{+}$ $CD64^{+}$ (A) and the expression of Ly6C and MHCII by Lin^{-} $CD11b^{+}$ $CD64^{+}$ myeloid cells has been assessed (B). Data are representative of three independent experiments. Bars indicate mean ± SEM. Statistical significance was assessed by non-parametric Mann-Whitney test. P<0.001 (***) and P<0.0001 (****) were considered statistically significant.

Figure S2. LPS-induced Mo-APCs are IL-18- and Asc-independent. A. As in Figure 1, Wild-type (WT), *Ifnar*1-KO, and *Caspase-1/11*-KO mice were injected intravenously (IV) with a non-lethal dose of ultrapure LPS from *E. coli* O111:B4 (25 µg/mouse), or PBS. IL-18 ELISA were done on blood serum collected 24h after LPS injection. B. As described in Figure 5, LPS ultrapure from *E. coli* O111:B4 (100 ng/ml) were added or not at day 0 in Flt3-L-dependent in vitro dendritic cells cultures generated with WT or *Pycard*-KO bone marrow cells. After 7 days, the Flt3-L-treated bone marrow cells were analyzed by flow cytometry for the presence of Mo-APC (gated as $MHCII^{+}$ $CD11c^{+}$ $CD64^{+}$) (left panel). The frequency of

this population was calculated for each condition (right panel). Data are representative of at least 2 independent experiments (A) and of at least 3 donor bone marrow mice done in quadruplicate. Bars indicate mean ± SEM. Statistical significance was assessed by one-way ANOVA/Bonferroni posttest. $P<0.05$ (*), $P<0.01$ (**), $P<0.001$ (***) and $P<0.0001$ (****) were considered statistically significant.

Figure S3. Decrease of blood monocytes numbers during endotoxemia. WT and *Ifnar1*-KO mice were treated as described in Figure 1. Blood was collected 24h after LPS injection and blood cells were analyzed by flow cytometry. Blood monocytes were gated as SSC^{low} FSC^{low} Lin^- $Ly6C^+$ $CD11b^+$ and their numbers per blood ml was calculated. Bars indicate mean ± SEM.

Figure S4. LPS-induced LSK cells proliferation is type I IFN independent. WT and *Ifnar1*-KO mice were treated as described in Figure 1. Bone marrows were collected 24h after LPS injection and analyzed by flow cytometry. Bone marrow LSK cells were gated as Lin^- $CD135^-$ $Ly6C^-$ $CD11b^-$ $MHCII^-$ $CD11c^-$ $CD117^+$ $Sca1^+$ (A and B) and their proliferation was assessed by in vitro BrdU incorporation (B). Plots are representative of three independent experiments done in triplicate (A). Bars indicate mean ± SEM from 3 independent experiments (B). Statistical significance was assessed by non-parametric Mann-Whitney test. $P<0.05$ (*), $P<0.01$ (**), $P<0.001$ (***) and $P<0.0001$ (****) were considered statistically significant.

Figure S5. Ifnar expression on monocyte precursors is required for LPS-induced Mo-APCs. A. Transcript levels of *Ifnar1* (left panel), and *Ifnar2* (righ panel) gene measured in MDP, cMoP, and monocytes extracted from the bone marrow of untreated wild-type mice. B. As described in Figure 6, WT or *Ifnar1*-KO $CD45.2^+$ MDP (gated as described in Figure 4) or cMop (gated as described in Figure 3) sorted by flow cytometry were co-cultured with $CD45.1^+$ bone marrow filler cells at day 0 in Flt3-L-dependent dendritic cells cultures.

Cultures were supplemented with LPS (100ng/ml), IFNα (100ng/ml) or IFNβ (10ng/ml) at day 0 and the DC and Mo-APC composition after 7 days was measured by flow cytometry among either the progeny of precursor cells or filler cells, the later used as an internal control (left panel). The frequencies of each population among the precursor-derived $CD45.2^+$ $CD11c^+$ $MHCII^+$ or filler-derived $CD45.1^+$ $CD11c^+$ $MHCII^+$ cells were calculated (right panel). Data are representative of at least 3 independent experiments (A, B) done in quadruplicate (B). Bars indicate mean ± SEM. Statistical significance was assessed by one-way ANOVA/Bonferroni posttest. $P<0.05$ (*), $P<0.01$ (**), $P<0.001$ (***) and $P<0.0001$ (****) were considered statistically significant.

Figure S1

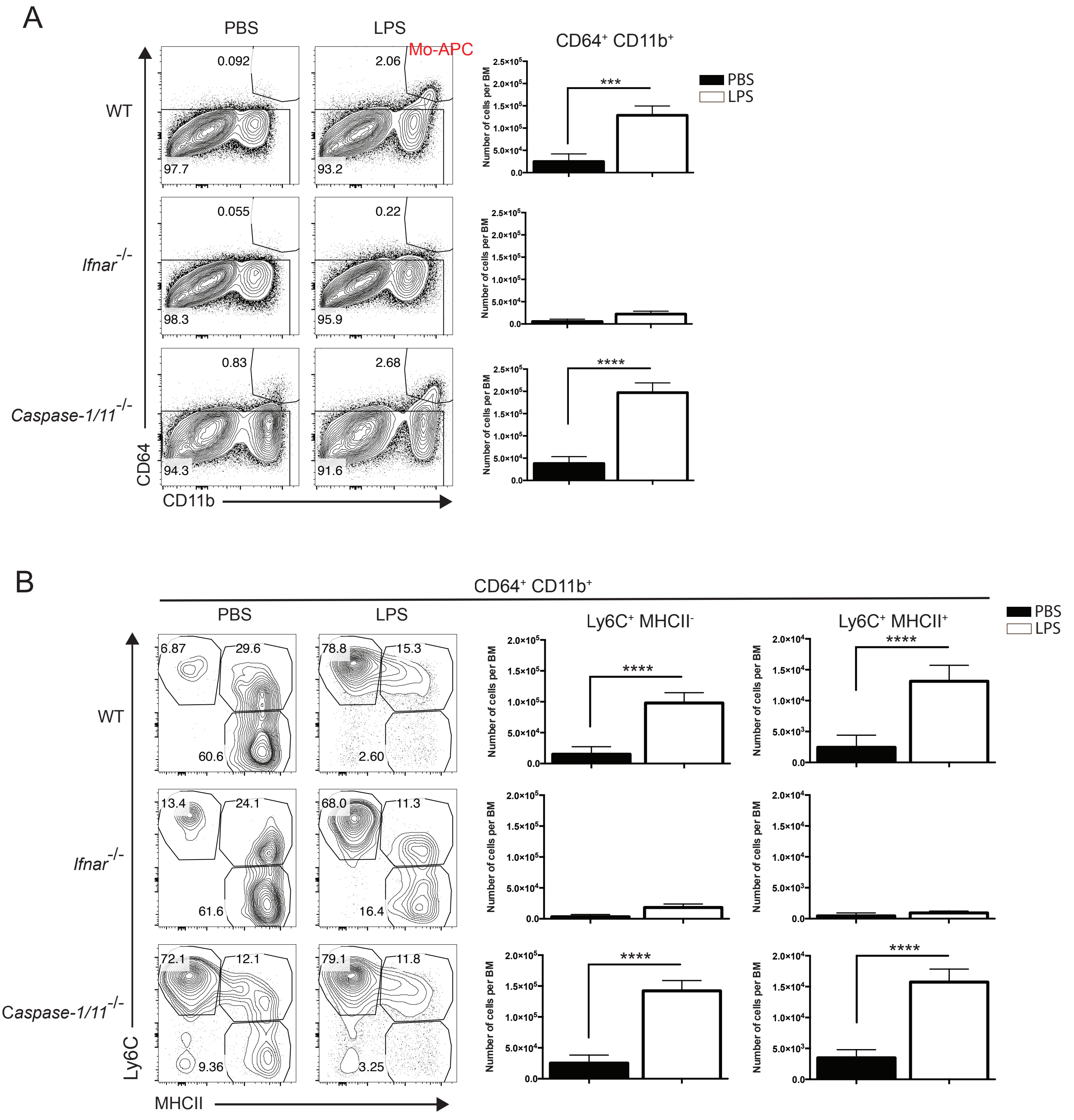

A
PBS
LPS
Mo-APC
CD64⁺ CD11b⁺
WT
Ifnar-/-
Caspase-1/11-/-
0.092
2.06
97.7
93.2
0.055
0.22
98.3
95.9
0.83
2.68
94.3
91.6
CD64
CD11b
Number of cells per BM
***
****
PBS
LPS
B
CD64⁺ CD11b⁺
Ly6C⁺ MHCII⁻
Ly6C⁺ MHCII⁺
6.87
29.6
60.6
78.8
15.3
2.60
13.4
24.1
61.6
68.0
11.3
16.4
72.1
12.1
9.36
79.1
11.8
3.25
Ly6C
MHCII

Figure S2

A

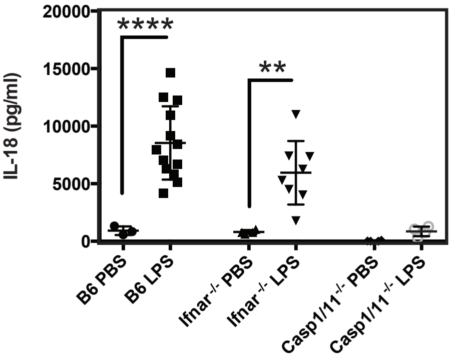

IL-18 (pg/ml)
20000
15000
10000
5000
0
****
**
B6 PBS
B6 LPS
Ifnar-/- PBS
Ifnar-/- LPS
Casp1/11-/- PBS
Casp1/11-/- LPS

B

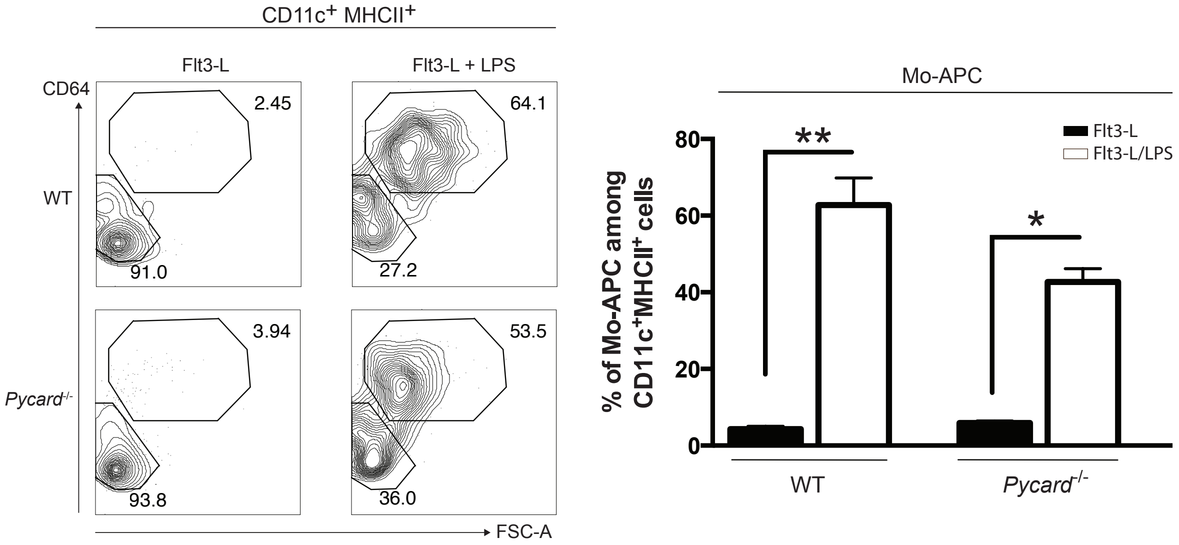

CD11c+ MHCII+
Flt3-L
Flt3-L + LPS
CD64
WT
Pycard-/-
2.45
91.0
64.1
27.2
3.94
93.8
53.5
36.0
FSC-A
Mo-APC
Flt3-L
Flt3-L/LPS
% of Mo-APC among CD11c+MHCII+ cells
80
60
40
20
0
**
*
WT
Pycard-/-

Figure S3

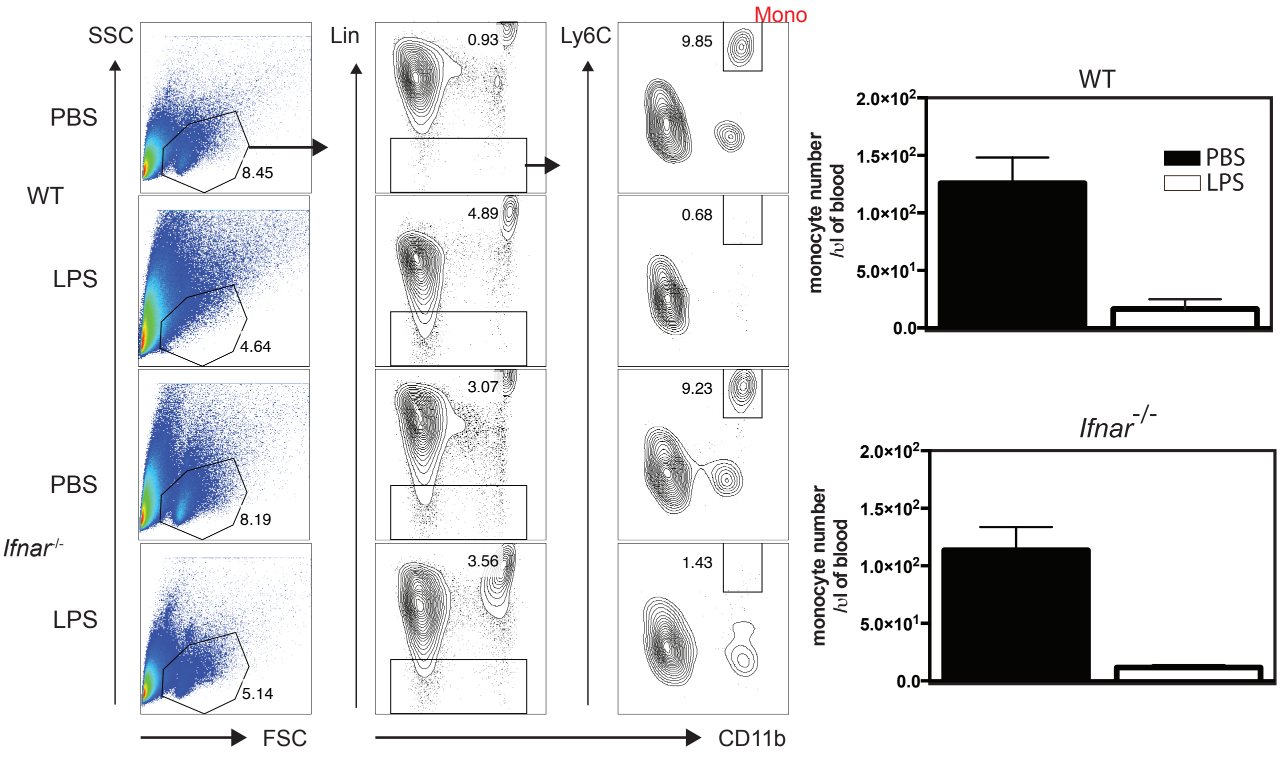

Mono
SSC
Lin
Ly6C
PBS
WT
LPS
PBS
Ifnar-/-
LPS
8.45
4.64
8.19
5.14
0.93
4.89
3.07
3.56
9.85
0.68
9.23
1.43
FSC
CD11b
WT
monocyte number /µl of blood
2.0×10^2
1.5×10^2
1.0×10^2
5.0×10^1
0.0
PBS
LPS
Ifnar-/-
monocyte number /µl of blood
2.0×10^2
1.5×10^2
1.0×10^2
5.0×10^1
0.0

Figure S4

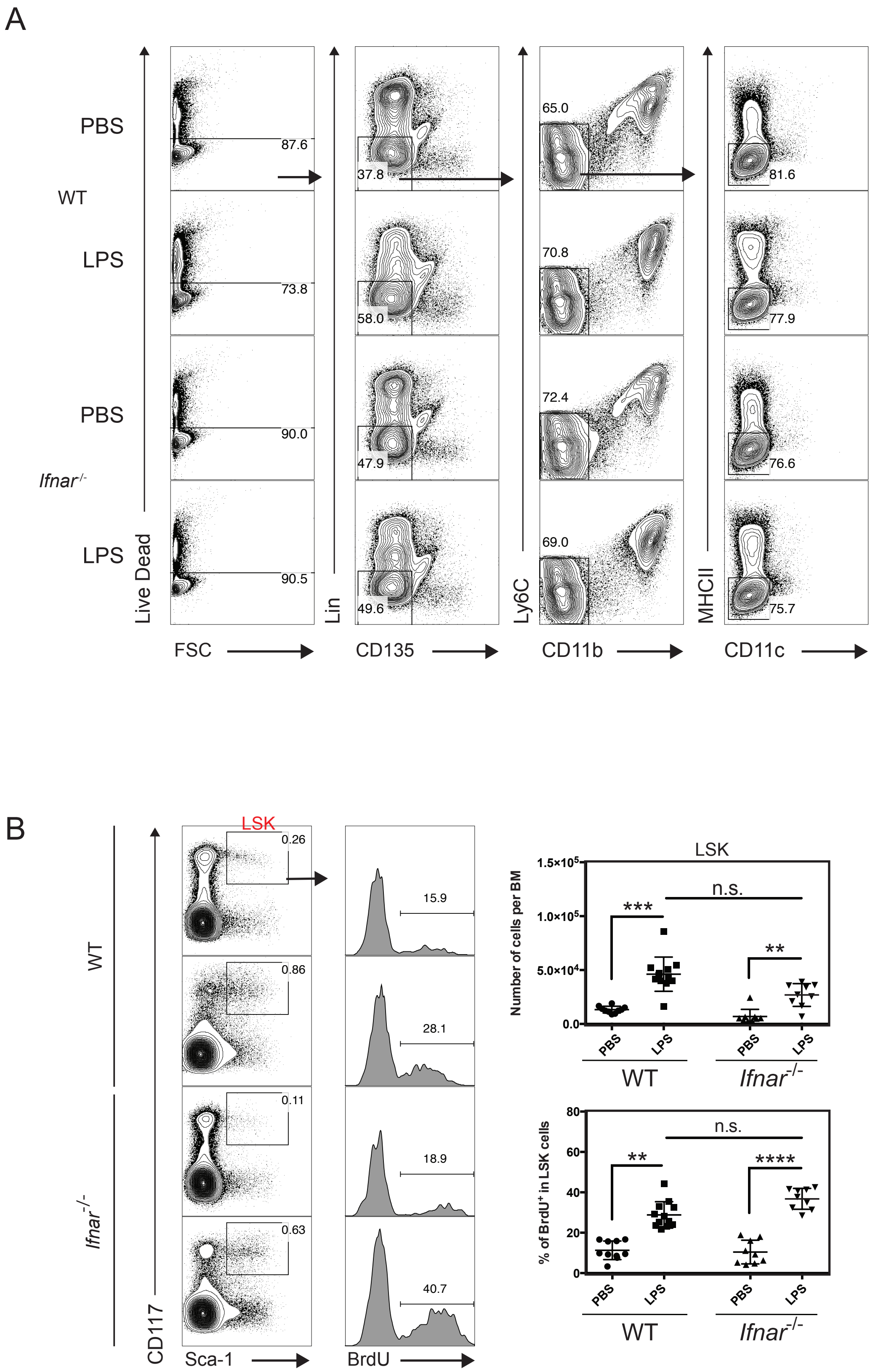
A
PBS
WT
LPS
PBS
Ifnar-/-
LPS
Live Dead
FSC
87.6
73.8
90.0
90.5
Lin
CD135
37.8
58.0
47.9
49.6
Ly6C
CD11b
65.0
70.8
72.4
69.0
MHCII
CD11c
81.6
77.9
76.6
75.7
B
WT
Ifnar-/-
LSK
0.26
0.86
0.11
0.63
CD117
Sca-1
15.9
28.1
18.9
40.7
BrdU
LSK
Number of cells per BM
1.5×10^5
1.0×10^5
5.0×10^4
0.0
***
**
n.s.
PBS
LPS
WT
Ifnar-/-
% of BrdU+ in LSK cells
80
60
40
20
0
**
****
n.s.

Figure S5

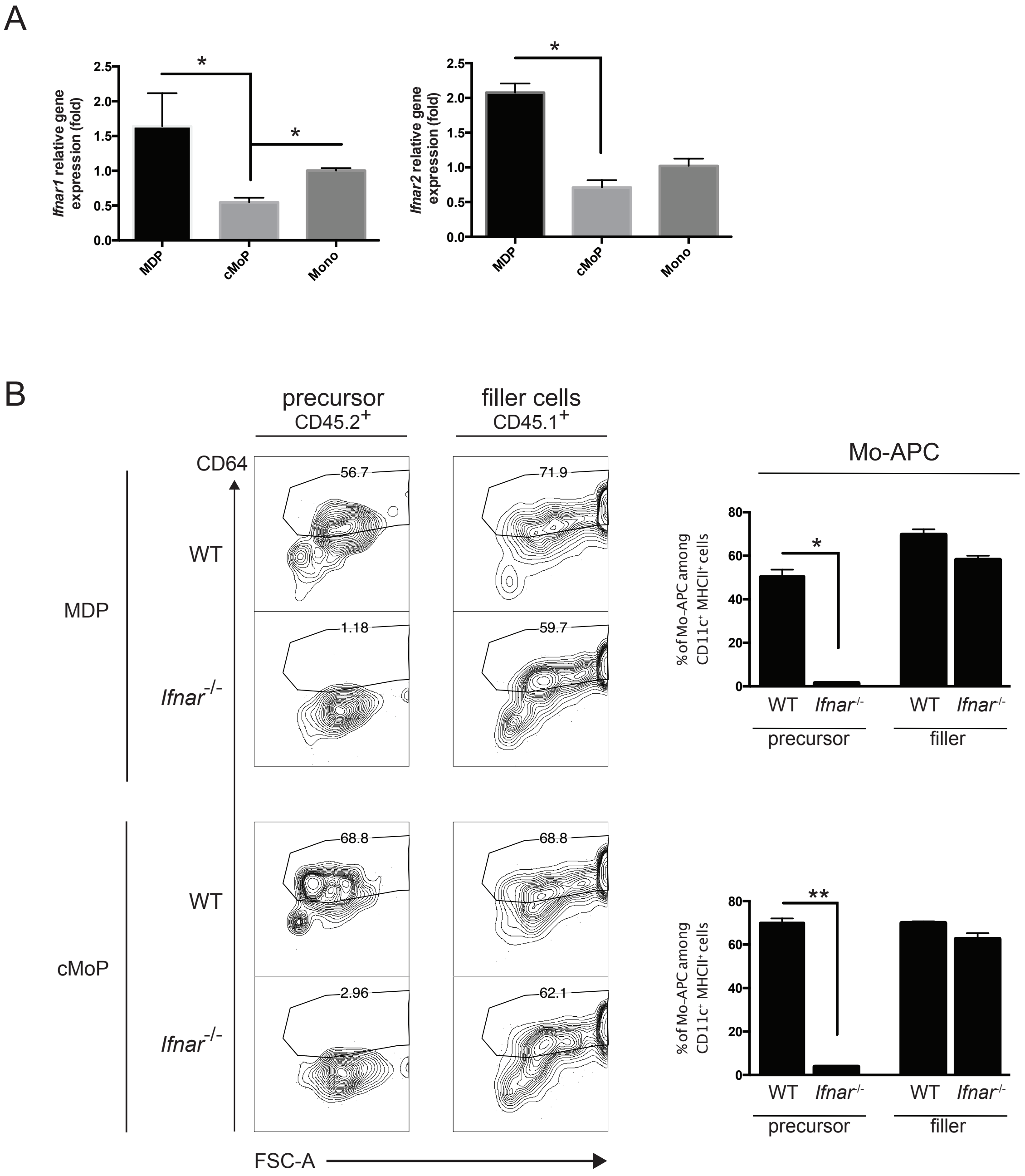

A
Ifnar1 relative gene expression (fold)
2.5
2.0
1.5
1.0
0.5
0.0
MDP
cMoP
Mono
*
*
Ifnar2 relative gene expression (fold)
B
precursor
CD45.2+
filler cells
CD45.1+
CD64
FSC-A
MDP
cMoP
WT
Ifnar-/-
56.7
71.9
1.18
59.7
68.8
68.8
2.96
62.1
Mo-APC
% of Mo-APC among CD11c+ MHCII+ cells
80
60
40
20
0
WT
Ifnar-/-
precursor
filler
**
